# 3T3-L1 Preadipocytes Exhibit Heightened Monocyte-Chemoattractant Protein-1 Response to Acute Fatty Acid Exposure

**DOI:** 10.1371/journal.pone.0099382

**Published:** 2014-06-09

**Authors:** Aimee L. Dordevic, Nicky Konstantopoulos, David Cameron-Smith

**Affiliations:** 1 School of Exercise and Nutrition Sciences, Deakin University, Melbourne, Victoria, Australia; 2 Department of Nutrition and Dietetics, Monash University, Notting Hill, Victoria, Australia; 3 Metabolic Research Unit, School of Medicine, Deakin University, Geelong, Victoria, Australia; 4 Liggins Institute, University of Auckland, Auckland, New Zealand; Universidade de Sao Paulo, Brazil

## Abstract

Preadipocytes contribute to the inflammatory responses within adipose tissue. Whilst fatty acids are known to elicit an inflammatory response within adipose tissue, the relative contribution of preadipocytes and mature adipocytes to this is yet to be determined. We aimed to examine the actions of common dietary fatty acids on the acute inflammatory and adipokine response in 3T3-L1 preadipocytes and differentiated mature adipocytes. Gene expression levels of key adipokines in 3T3-L1 preadipocytes and adipocytes were determined following incubation with palmitic acid, myristic acid or oleic acid and positive inflammatory control, lipopolysaccharide for 2 and 4 h. Inflammatory kinase signalling was assessed by analysis of nuclear factor-κB, p38-mitogen-activated protein kinase and c-jun amino-terminal kinase phosphorylation. Under basal conditions, intracellular monocyte chemoattractant protein-1 and interleukin-6 gene expression levels were increased in preadipocytes, whereas mature adipocytes expressed increased gene expression levels of leptin and adiponectin. Fatty acid exposure at 2 and 4 h increased both monocyte chemoattractant protein-1 and interleukin-6 gene expression levels in preadipocytes to greater levels than in mature adipocytes. There was an accompanying increase of inhibitor of κB-α degradation and nuclear factor-κB (p65) (Ser536) phosphorylation with fatty acid exposure in the preadipocytes only. The current study points to preadipocytes rather than the adipocytes as the contributors to both immune cell recruitment and inflammatory adipokine secretion with acute increases in fatty acids.

## Introduction

Adipose tissue is broadly comprised of two fractions, mature adipocytes and the stromal vascular fraction (SVF) [1]–[4], where the latter generates many of the pro-inflammatory factors secreted from adipose tissue [1]. Within the SVF, preadipocytes, which are the undifferentiated precursors of mature adipocytes, account for 15 to 50% of cells in human adipose tissue [5]. Preadipocytes share numerous phenotypic features with pro-inflammatory macrophages [6], [7] including the capacity to secrete inflammatory mediators such as TNFα, MCP-1, and IL-6 [8]. The propensity for greater inflammatory response in preadipocytes is mediated by the nuclear factor-κB (NF-κB) and mitogen-activated protein kinases (MAPK) such as c-jun amino-terminal kinase (JNK) signalling in preadipocyte cells compared with mature adipocytes [9].

Cytokine secretion from adipose tissue is acutely influenced by the macronutrient composition of a meal and further by the lipid species present within high-fat meals [10]. In the immediate hours following a meal, key metabolic adaptations occur in conjunction with inflammatory changes throughout the body. Dysregulation of acute metabolic adaptations occur in people with chronic metabolic disorders. Inflammatory markers such as TNFα, IL-6 and ICAM-1 are increased in healthy individuals, but are reported to be higher in T2D patients after 4 hours following a high fat meal [11]. Following the consumption of high saturated fatty acid (SFA), monounsaturated fatty acid (MUFA) or polyunsaturated fatty acid (PUFA) milkshakes, overweight and obese adults exhibit increased plasma CRP levels for up to 6 hours, with no difference between FA composition of the beverage, whereas TNFα and VCAM levels remain stable. However, ICAM levels were observed to be reduced following consumption of the MUFA meal compared with SFA and PUFA meals, indicating the importance of the FA composition in postprandial regulation of inflammation [12].

SFA, are potent activators of toll-like receptors (TLR) [13] that activate NF-κB [14] and p38-MAPK [15], [16] signalling, eliciting pro-inflammatory cytokine generation. These actions have been demonstrated in mature adipocytes, typically following sustained fatty acid (FA) exposure (>6 h) [17], [18]. However, there is minimal data available on the differences in inflammatory cytokine expression and activation of NF-κB and MAPK stress-signalling kinase pathways in preadipocytes compared with mature adipocytes following acute (≤4 h) exposure to FA, mimicking heightened concentrations of a single high-fat meal. The current study therefore aimed to analyse the impact of individual common dietary FAs, including the predominant saturated species, myristic and palmitic acids (C14∶0 and C16∶0, respectively) and the predominate MUFA, oleic acid (C18∶1), which is assumed to exert minimal impact on postprandial inflammation [19], [20]. It was hypothesised that in response to SFA exposure and in contrast to MUFA exposure, preadipocytes would generate an inflammatory response that was attenuated in mature adipocytes.

## Methods

### 3T3-L1 Cell culture

3T3-L1 fibroblasts (American Type Culture Collection (ATCC) and as detailed in [21]), were cultured to 2 days post-confluence in 5% CO_2_ using high glucose (4.5 g/L) D-Modified Eagle's Medium (DMEM) supplemented with 10% (v/v) fetal bovine serum (FBS) and 1% (v/v) penicillin/streptomycin to yield preadipocytes. Adipocytes were differentiated in DMEM supplemented with 10% (v/v) FBS, 2 µg/ml Humulin human insulin (Eli Lilly Australia, West Ryde, NSW, Australia), 0.25 µM dexamethasone (Sigma Aldrich, Castle Hill, NSW, Australia) and 0.5 mM 3-isobutyl-1-methylxanthine (IBMX) (Sigma Aldrich) for 3 days. Subsequently, adipocytes were maintained in post-differentiation DMEM with 2 µg/ml insulin for a further 3 days then replenished with DMEM with 5% (v/v) FBS and 1% (v/v) penicillin/streptomycin for at least 24 h before treatments. Preadipocytes and adipocytes were serum-starved in DMEM with 1% (v/v) penicillin/streptomycin supplemented with 0.2% (w/v) fatty acid (FA)-free BSA (low endotoxin–grade, Sigma Aldrich) for 2 h prior to all treatments. All experiments were performed on 5 independent occasions. The mRNA and protein levels of preadipocyte factor-1 (Pref-1), a marker of pre-differentiation [22], were increased by 19- (p<0.0001) and 3-fold (p = 0.008), respectively, in the preadipocyte cell populations compared with the mature adipocytes (Figure S1).

### Fatty acid treatment preparation

FA were prepared by a method adapted from Chavez et al. [23]. Briefly, FA were dissolved in ethanol, then diluted to the required concentrations (0.1, 0.25 or 0.5 mM) in 2% (w/v) FA-free BSA (low endotoxin-grade) in DMEM with 1% (v/v) penicillin/streptomycin and conjugated overnight at 37°C until dissolved. Treatment and vehicle control ethanol concentrations were normalised to 0.17% (v/v). Cells were treated for 2 and 4 h in DMEM containing 2% (w/v) FA-free BSA and 1% (v/v) penicillin/streptomycin with or without FA. Following treatment, cells were washed twice with ice-cold 1× phosphate-buffered saline (PBS), pH 7.4 (Life Technologies) prior to RNA and protein extraction. Lipopolysaccharide (LPS) at 10 ng/ml was used as a positive control for the measurement of inflammatory adipokines. Dose response experiments were conducted with 0.1 0.25 and 0.5 mM for each FA at 37°C. The final concentration used for all subsequent analysis was 0.5 mM as it consistently induced an inflammatory response (data not shown).

### Gene expression levels of inflammatory mediators and adipokines

Cells were collected after 2 and 4 h in TRI Reagent (Life Technologies Corporation). Total RNA concentration and quality were determined using the Nanodrop ND-1000 (Nanodrop Technologies, Delaware, USA). First strand cDNA was generated from 0.5 µg total RNA using the High Capacity RNA-to-cDNA kit (Life Technologies Corporation). Analysis of gene expression was performed with the CFX384 Real-Time PCR Detection System (Bio Rad Laboratories, Hercules, CA, USA) and HOT FIREPol EvaGreen qPCR Mix Plus (ROX) (Bio Rad Laboratories) using gene-specific primers designed using Primer Express 3.0 (Life Technologies Corporation) software (refer to Table S1 for the list of primer sequences). Each sample was analysed in duplicate with positive and negative controls. Data was normalised to acidic ribosomal phosphoprotein (36B4), an unaltered gene during adipogenesis [24]. Data were analysed using a comparative critical threshold (Ct) method, where the amount of target gene normalised to the amount of endogenous control relative to control value is given by 2^−ΔΔCt^.

### Phosphorylation and protein levels of key stress kinases

Cells were harvested for measurement of signalling kinases in total cellular protein at 1 and 2 h in RIPA lysis buffer (Millipore, Billerica, MA, USA) with freshly added protease and phosphatase inhibitors PMSF (1 mM), Na_3_VO_4_ (1 mM), NaF (1 mM), aprotinin (1 µg/ml) and leupeptin (1 µg/ml). Protein concentration was determined using the BCA protein assay kit (Thermo Scientific Inc., Scoresby, VIC, Australia) and whole cellular lysates were diluted in RIPA lysis buffer to obtain equivalent protein concentrations between samples. Thirty micrograms of protein lysates were resolved via 8-10% SDS-PAGE gels under reducing conditions (MP3 Mini Protean Gel system, Bio-Rad Laboratories) and then transferred onto nitrocellulose membranes (Bio-Rad Laboratories) using Towbin's transfer buffer, pH 8.3 and blocked in 5% (w/v) bovine serum albumin (BSA) (Sigma Aldrich) prepared in 1× Tris buffered saline, pH 7.5 with 1% Tween20 (TBST), and incubated with the relevant primary antibody phospho-NF-κB (p65) (Ser536), IκBα, phospho p38 (Thr180/Tyr182) or phospho JNK (Thr183/Tyr185) (Cell Signalling, Danvers, MA, USA) at 1∶1000 in 5% (w/v) BSA in TBST overnight at 4°C. Membranes were incubated with horse radish peroxidise (HRP)-conjugated secondary antibody (at 1∶2000), in 5% (w/v) BSA in TBST for 2 h at room temperature. Bands were detected using Western Lighting Chemiluminescence Reagent Plus (Perkin Elmer Life Sciences Inc, MA, USA) and images collected on a Kodak Digital Science Image Station (Model 440CF, Eastman Kodak Company, Rochester, NY, USA). Densitometry readings were obtained using Kodak Molecular Imaging Software (Version 4.0.5, Eastman Kodak Company). Subsequently, the membranes were re-probed with the required antibodies to detect total protein; p65, p38 and SAPK/JNK (Cell Signaling, Danvers, MA, USA) or α-tubulin (Santa Cruz Biotechnology Inc., Santa Cruz, CA, USA) as a loading control.

### Statistical analysis

Statistical analysis was conducted using Statistical Package for the Social Sciences (SPSS version 20.0 Fullerton, CA, USA). Baseline expression values were compared using unpaired t-tests. Homogeneity of variance was determined using Levene's Test and post-hoc analyses of two-way ANOVA were performed as t-tests with Bonferroni adjustment for multiple comparisons. Pair-wise comparisons were performed for analysis of main effects. Data were considered as significant at p<0.05. All results have been represented as mean ±SEM, where each experiment was performed 5 independent times (n = 5), unless otherwise specified.

## Results

### Intracellular MCP-1 and IL-6 gene expression levels were increased in preadipocytes compared with mature adipocytes before and after FA exposure

**Figure 1:**
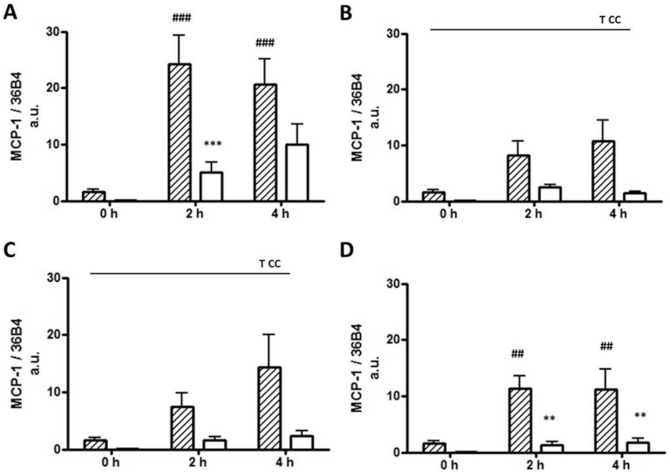
MCP-1 mRNA levels in 3T3-L1 preadipocytes and adipocytes. MCP-1 gene expression levels of 3T3-L1 preadipocytes (hatched bars) and adipocytes (open bars) treated with (A) LPS (10 ng/ml); (B) Palmitic acid (0.51 mM); (C) Myristic acid (0.5 mM); and (D) Oleic acid (0.5 mM) at 0, 2 and 4 h. Data are presented as mean ±SEM (*n* = 5) normalised to 36B4. ** p<0.01, *** p<0.001 versus preadipocytes, ^##^ p<0.01, ^###^ p<0.001 versus 0 h. Main time effect T p<0.05, main cell type effect CC p<0.01.

**Figure 2:**
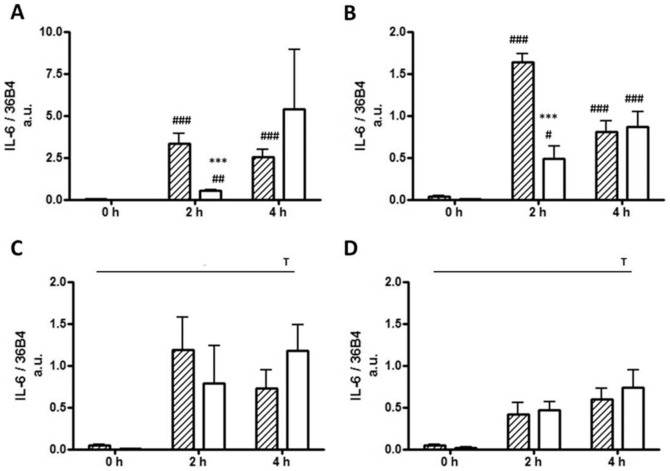
IL-6 mRNA levels in 3T3-L1 preadipocytes and adipocytes. IL-6 gene expression levels of 3T3-L1 preadipocytes (hatched bars) and adipocytes (open bars) treated with (A) LPS (10 ng/ml); (B) Palmitic acid (0.5 mM); (C) Myristic acid (0.5 mM); and (D) Oleic acid (0.5 mM) at 0, 2 and 4 h. Data are presented as mean ±SEM (*n* = 5) normalised to 36B4. *** p<0.001 versus preadipocytes, ^#^ p<0.05, ^##^ p<0.01, ^###^ p<0.001 versus 0 h. Main time effect T p<0.05.

At baseline (0 h) and prior to FA exposure, preadipocytes exhibited elevated levels of MCP-1 gene expression (22-fold, p<0.0001) compared with mature adipocytes. MCP-1 gene expression levels were increased from baseline in both adipocytes and preadipocytes following incubation with LPS (p = 0.0002), yet remained ≥2.1-fold higher in preadipocytes compared to adipocytes (p = 0.0006). Palmitic and myristic acids increased MCP-1 expression levels in both cell types (p≤0.025), however, expression levels were ≥3.3-fold higher in preadipocytes compared with mature adipocytes at each time point (p = 0.002 and p = 0.005 respectively, Figure 1B and C). Oleic acid also resulted in increased MCP-1 gene expression levels in both cell types and preadipocytes exhibited a ≥6.2-fold increase in expression levels compared with mature adipocytes at 2 and 4 h (p≤0.047) (Figure 1D). Similarly, IL-6 mRNA levels were increased 7.7-fold in preadipocytes compared with mature adipocytes at baseline (p<0.0001). IL-6 mRNA levels increased in both cell types over time with all treatments; LPS (Figure 2A; p<0.0001); palmitic acid (Figure 2B; p<0.0001); myristic acid (Figure 2C; p = 0.012) and oleic acid (Figure 2D; p = 0.001). Both LPS (6.6-fold, p = 0.007) and palmitic acid (3.4-fold, p<0.0001) induced higher IL-6 expression levels in the preadipocytes compared to the adipocytes at 2 h.

**Figure 3:**
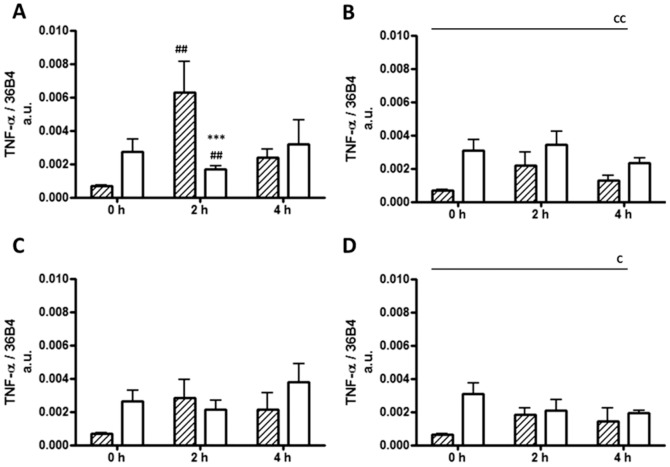
TNFα mRNA levels in 3T3-L1 preadipocytes and adipocytes. TNFα mRNA expression of 3T3-L1 preadipocytes (hatched bars) and adipocytes (open bars) treated with (A) LPS (10 ng/ml); (B) Palmitic acid (0.5 mM); (C) Myristic acid (0.5 mM); and (D) Oleic acid (0.5 mM) at 0, 2 and 4 h. Data are presented as mean ±SEM (*n* = 5) normalised to 36B4. *** p<0.001 versus preadipocytes, ^##^ p<0.01 versus 0 h. Main cell type effect C p<0.05, CC p<0.01.

TNF-α gene expression levels were lower in preadipocytes at baseline compared with mature adipocytes (p = 0.003, Figure 3A–D). With exposure to the positive control LPS, there was an acute and transient 9.2-fold increase at 2 h in TNF-α expression levels in the preadipocytes, which was absent in mature adipocytes (p = 0.028, Figure 3A). None of the 3 FA affected the gene expression levels of intracellular TNF-α at 2 or 4 h in either cell type (Figure 3B-D). Both leptin (10-fold, p<0.0001) and adiponectin (843-fold, p<0.0001) gene expression levels were increased in mature adipocytes compared with preadipocytes (Figure S2 and S3), however, no changes in gene expression levels were observed in response to any treatment.

### Acute fatty acid exposure induced IκBα degradation and NF-κB (p65) phosphorylation, but not p38-MAPK and JNK phosphorylation, in preadipocytes

**Figure 4:**
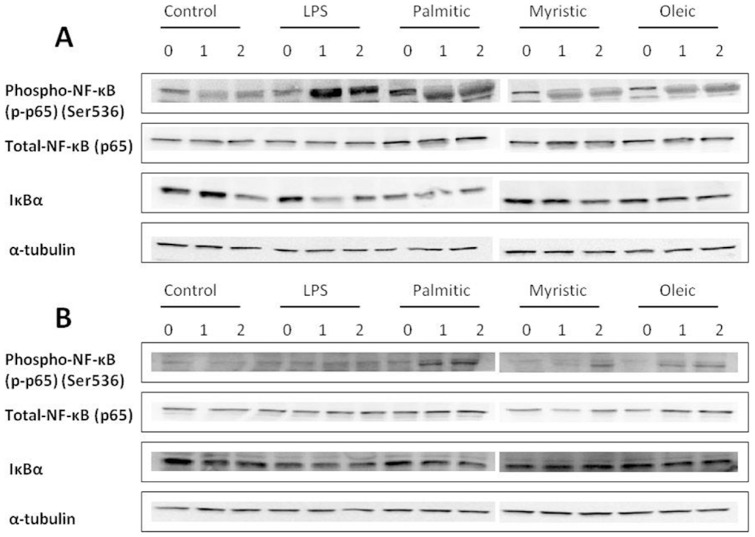
Representative immunoblots of NF-κB activation in 3T3-L1 preadipocytes and mature adipocytes. 3T3-L1 cells were treated with LPS (10 ng/ml); Palmitic acid (0.5 mM); Myristic acid (0.5 mM); and Oleic acid (0.5 mM) for 0, 1 and 2 h. Phosphorylation levels of p65 (Ser536) relative to total p65; and total levels of IκBα relative to α-tubulin were measured by Western blot analysis in 3T3-L1 (A) preadipocytes and (B) mature adipocytes (n = 5).

Levels of NF-κB (p65) phosphorylation on Ser536 were increased in preadipocytes treated with LPS by 2.8-fold and 1.9-fold at 1 and 2 h (p = 0.002), respectively, and myristic acid by 2.2-fold at 1 h and 2.1-fold at 2 h (p = 0.012) compared with vehicle-treated preadipocytes (Figure 4A). In contrast, increased NF-κB phosphorylation by palmitic acid (1.9-fold at 1 and 2 h, p = 0.074) and oleic acid (1.6-fold at 1 h and 1.7-fold at 2 h, p = 0.459) was not observed. IκBα, an inhibitory binding partner of cytosolic NF-κB, was reduced in preadipocytes following treatment with positive control LPS by 0.7-fold at 1 and 2 h (p = 0.04), palmitic acid by 0.7-fold at 1 h and 0.6-fold at 2 h (p<0.0001) and myristic acid 0.8-fold at 1 and 2 h (p = 0.019) compared with vehicle-treated cells (1.1-fold at 1 and 2 h compared with baseline, 0 h) (Figure 4A). As observed with NF-κB phosphorylation, oleic acid had no effect on IκBα protein levels (Figure 4A). Mature adipocytes demonstrated a 1.3 to 1.4-fold increase in NF-κB (p65) phosphorylation over time (p = 0.005) with no difference between treatments (Figure 4B). No changes were observed in IκBα abundance in mature adipocytes following any treatment (Figure 4B). Phospho NF-κB (p65) (Ser536) and IκBα densitometry analyses are shown in Figure S4.

**Figure 5:**
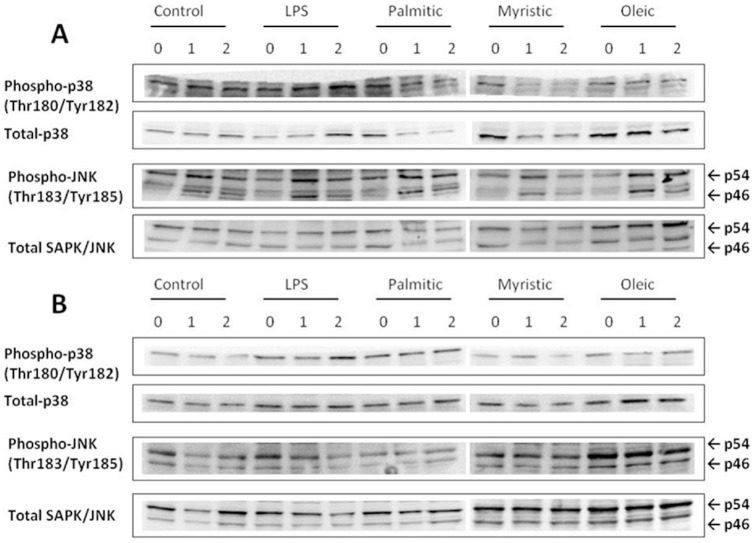
Representative immunoblots of MAPK phosphorylation in 3T3-L1 preadipocytes and mature adipocytes. 3T3-L1 cells were treated with LPS (10 ng/ml); Palmitic acid (0.5 mM); Myristic acid (0.5 mM); and Oleic acid (0.5 mM) for 0, 1 and 2 h. Phosphorylation levels of p38 (Thr180/Tyr182) relative to total p38; and phosphor-JNK (Thr183/Tyr185) relative to total JNK were measured by Western blot analysis in 3T3-L1 (A) preadipocytes and (B) mature adipocytes (n = 5).

Levels of p38-MAPK (Thr180/Tyr182) phosphorylation were unaffected in preadipocytes following all FA treatments at 1 and 2 h (Figure 5A). An effect on p38-MAPK (Thr180/Tyr182) phosphorylation was only measured with LPS, which increased phosphorylation by 1.4-fold compared with vehicle-treated cells at 2 h in the mature adipocytes (p = 0.029) (Figure 5B). A main time effect was observed for JNK (p54) phosphorylation levels, which increased at least 1.1-fold at 1 h (p = 0.001) with all treatments in the preadipocytes (Figure 5A). Phosphorylation of JNK (p46) was increased ≥2.6-fold with time (p<0.0001) compared with baseline in the preadipocytes, however no significant differences were found between any of the FA treatments (Figure 5A). There were no differences in JNK phosphorylation in mature adipocytes with any of the FA treatments (Figure 5B). Phospho p38-MAPK (Thr180/Tyr182) and phospho JNK (p45, p54) (Thr183/Tyr185) densitometry analyses are shown in Figure S4.

## Discussion

Preadipocytes are a major cellular fraction present within white adipose tissue and are reported to significantly contribute to the production and secretion of inflammatory adipokines involved in the pathogenesis of chronic disease [25]. Several studies have reported the inflammatory nature of preadipocytes in response to stimuli such as LPS [9], [26] and macrophage-secreted factors [27], [28]. Despite evidence that SFA elicit inflammatory responses in adipose tissue [14], [29]–[33], the contribution of preadipocytes to this effect is far less understood. These actions may be important, notably in the post-meal period when increased FA occurs transiently for several hours [34]–[36]. In the present study, we demonstrated that FA exposure for 2 hours induced an inflammatory gene expression response, predominantly MCP-1 in the preadipocytes. These inflammatory responses were blunted in mature adipocytes. Analysis of key stress kinase signalling responses demonstrated selective activation of the NF-κB pathway, but not p38-MAPK or JNK in preadipocytes in response to the SFA; myristic and palmitic acids, and to the MUFA oleic acid.

Prior to FA treatments, 3T3-L1 preadipocytes exhibited increased baseline gene expression levels of both MCP-1 and IL-6 compared with mature adipocytes, as reported previously in this cell line and cultured human primary and 3T3-L1 cells [9], [26]. In contrast to the previous data in primary human preadipocytes [9], this study demonstrated increased TNF-α mRNA expression levels in the mature 3T3-L1 adipocytes. As expected differentiated and matured adipocytes express increased levels of leptin and adiponectin [37]. Furthermore, as described previously, adiponectin and leptin did not respond markedly to acute FA exposure [38]. Over extended time periods (24hrs plus) the adiponectin gene expression may be regulated by FA species, as oleic acid has been demonstrated to selectively increased adiponectin gene expression in 3T3-L1 adipocytes [39]. A key limitation of the current study was the absence of measuring the abundance of the intracellular and secreted transcribed proteins. Gene responses may only be a partial correlate of protein production and secretion. As an example, it has been reported that a lack of TNF-α converting enzyme (TACE) activity in preadipocytes, affects their ability to secrete TNF-α [6]. Hence, the observed increase in preadipocyte TNF-α gene expression levels with LPS may not reflect a functional response.

Acute treatment with FA induced a prominent increase in MCP-1 mRNA levels in preadipocytes. The present study did not measure cytokine protein levels, however, previous studies have identified that increased MCP-1 gene expression levels in preadipocytes is reflected by greater protein expression and secretion compared with mature adipocytes [6], [40]. A recent study demonstrated increased MCP-1 protein secretion from 3T3-L1 preadipocytes in response to 0.1 mM palmitic acid over 72 hours [41], highlighting that it is likely that changes in MCP-1 gene expression levels reported in the current study could translate to a functional response by the preadipocytes.

MCP-1 is a potent chemoattractant for macrophage infiltration and activation [42], [43]. Macrophages recruited to adipose tissue in response to a high fat diet, exhibit an inflammatory phenotype compared to resident macrophage populations [44]. Murine MCP-1 deficiency models display reduced adipose tissue macrophage accumulation [42]. Conversely, overexpression of MCP-1 results in increased adipose tissue macrophages and insulin resistance [43]. Furthermore, decreasing MCP-1 secretion from human preadipocytes has been shown to reduce monocyte migration *in vitro* [40]. This suggests that FA-induced MCP-1 expression in preadipocytes may contribute to adipose tissue macrophage accumulation observed in diet-induced obesity.

It was surprising to observe an increased MCP-1 response to oleic acid, which is classically considered to be FA with a predominant anti-inflammatory impact on adipose tissue [45]. Further, 0.16 mM oleic acid has recently been demonstrated to induce differentiation in chicken preadipocytes after 12 hours [46]. Despite this, 0.1 mM oleic acid has been shown to synergistically activate NF-κB when combined with adipocyte-conditioned medium in human vascular smooth muscle cells (SMC) [47]. Further, prolonged exposure with 0.5 mM oleic acid results in insulin resistance via increased p38-MAPK phosphorylation in primary hepatocytes [48]. Together with previous studies, the current study suggests that the role of oleic acid is dependent on concentration and site of exposure. While phosphorylation of p38-MAPK and JNK was not significantly increased at 1 or 2 h with oleic acid in the current study, there is the potential for crosstalk with NF-κB, and activation may occur prior or subsequent to phosphorylation of NF-κB (p65) in the preadipocytes (reviewed in [49]).

Acute FA treatments demonstrated only a modest increase in IL-6 and TNF-α gene expression levels in preadipocytes when compared with MCP-1. Our findings contrast previous chronic studies (24 to 48 h) in mature 3T3-L1 adipocytes that demonstrated increased MCP-1 [18], IL-6 [17] and TNF-α [33] gene expression levels via NF-κB activation with palmitic acid treatment. Another key SFA, stearic acid, has also been shown to exert pro-inflammatory effects after 24 hours [18], however the shorter chain SFA, lauric acid [17] and MUFA, oleic acid [18], [33] reportedly have no effect on these markers in mature adipocytes even over longer periods of time. Despite the link between myristic acid and chronic disease [50], there are surprisingly few studies, if any, which have examined the inflammatory effects of this FA *in vitro*. The present study demonstrated that myristic acid exposure also elicits an acute inflammatory cytokine response, particularly MCP-1, to the same extent as palmitic acid in preadipocytes.

Consistent with previous data [51], it was interesting to measure a lack of NF-κB activation via IκBα degradation and p65 phosphorylation in the mature adipocytes, even in the presence of LPS. This effect may be time-dependent, as sustained exposure (24 to 48 h) of 0.5 mM palmitic acid has been previously reported to increase NF-κB binding in 3T3-L1 adipocytes [52]. Yet, preadipocytes demonstrated a pronounced, acute activation of NF-κB by all FA species. It is not possible from the present data to determine whether this is a downstream event elicited directly by FA exposure, potentially via TLR activation, or alternatively as a consequence of heightened cytokine protein synthesis and receptor binding [6], [25], [37]. Our data indicate that palmitic, myristic and oleic acids do not acutely activate stress kinase signalling via phosphorylation of JNK or p38-MAPK over 4 hours. However, recent evidence indicates that the presence of 50 µM n-3 PUFA docosahexaenoic acid (DHA) during differentiation reduces phosphorylated p38 MAPK levels in matured adipocytes. Thus, the type and ratio of mixture of the fatty acids that are available to preadipocytes may be important in determining the inflammatory response.

Previous studies have explored the effect of mixed FA in adipocytes and have demonstrated increased JNK and IκB kinase (IKK) phosphorylation (the latter responsible for NF-κB activation) and reduced glucose uptake after 1 h [53] as well as increased gene expression levels of TLR2 gene after 8 hours [54], and TLR4 after 3 hours. It has also been recently demonstrated by Neacsu et al. that human preadipocytes, treated with mixed FFA, exhibit increased JNK phosphorylation at 20 minutes as well as elevated TNF-α and MCP-1 gene expression levels after 4 hours [55]. The aim of our study was to explore the effects of individual, common dietary fatty acids in order to compare their impact on preadipocytes and adipocytes. Our findings can guide future studies to determine FA mixtures that may elicit the most beneficial effect on inflammation in adipocytes and preadipocytes, and may lead to targeting individual FA's in the diet for therapeutic purposes.

In conclusion, the present study demonstrates that preadipocytes have a heightened inflammatory cytokine response following acute SFA and MUFA exposure, when compared to mature adipocytes. This effect was pronounced for MCP-1, a major chemoattractant responsible for macrophage infiltration and activation. Preadipocytes also demonstrated activation of the NF-κB pathway following FA exposure. The responsiveness of pro-inflammatory adipokines was not consistent, as TNF-α mRNA levels were not altered in either preadipocytes or adipocytes following acute FA exposure. From the current data, it can be concluded that preadipocytes exert a predominant role, via MCP-1, to macrophage recruitment in adipose tissue in the hours immediately following meal ingestion and contribute to postprandial inflammatory responses. Monocyte infiltration into adipose tissue and subsequent inflammatory polarization is a key phenomenon in obesity-related disorders [56], [57]. Previously, MCP-1 secretion from preadipocytes has been demonstrated to induce inflammatory monocyte migration *in vitro* [40] and future studies could explore this in response to FA. Given the complexity of adipose tissue, further investigation into potential crosstalk between the inflammatory responses elicited by preadipocytes and the other cell types present in adipose tissue, including mature adipocytes and monocytes is required.

## Supporting Information

Figure S1 Preadipocyte factor-1 (pref-1) expression in 3T3-L1 preadipocytes and adipocytes. (A) mRNA and (B) protein expression of pref-1 in 3T3-L1 preadipocytes (2 days post-confluence) (hatched bars) and adipocytes (open bars) differentiated 6 days then maintained for a further 24 h (n = 5). ** p<0.01, *** p<0.001.
(TIF)

Figure S2 Leptin mRNA expression in 3T3-L1 preadipocytes and adipocytes. Leptin mRNA expression of 3T3-L1 preadipocytes (hatched bars) and adipocytes (open bars) treated with (A) LPS (10 ng/ml); (B) Palmitic acid (0.5 mM); (C) Myristic acid (0.5 mM); and (D) Oleic acid (0.5 mM) at 0, 2 and 4 h. Data are presented as mean ±SEM (*n* = 5) normalised to 36B4. * p<0.05, *** p<0.001 versus preadipocytes, ^##^ p<0.01 versus 0 h. Main effects, Main cell type effect CC p<0.01, CCC p<0.001.
(TIF)

Figure S3 Adiponectin mRNA expression in 3T3-L1 preadipocytes and adipocytes. Adiponectin mRNA expression of 3T3-L1 preadipocytes (hatched bars) and adipocytes (open bars) treated with (A) LPS (10 ng/ml); (B) Palmitic acid (0.5 mM); (C) Myristic acid (0.5 mM); and (D) Oleic acid (0.5 mM) at 0, 2 and 4 h. Data are presented as mean ±SEM (*n* = 5) normalised to 36B4. Main cell type effect CCC p<0.001.
(TIF)

Figure S4 3T3-L1 adipocytes were treated with LPS (10 ng/ml); Palmitic acid (0.5 mM); Myristic acid (0.5 mM); and Oleic acid (0.5 mM) for 0 (open bars), 1 (grey bars) and 2 h (black bars). Phosphorylation levels of p65 (Ser536) relative to total p65 and IκBα relative to α-tubulin in (A) and (C) preadipocytes and; (B) and (D) adipocytes, respectively. Phosphorylation levels of p38 MAPK (Thr180/Tyr182) relative to total p38 MAPK in (E) preadipocytes and (F) adipocytes. Phosphorylation levels of JNK (Thr183/Tyr185) relative to total JNK, p54 in (G) preadipocytes and (H) adipocytes and; p46 in (I) preadipocytes and (J) adipocytes, respectively as measured by Western blot analysis. Data are presented as fold change mean ±SEM (*n* = 5). Significant interactions, * p<0.05, ** p<0.01, *** p<0.001 versus control. Main time effects, TT p<0.01, TTT p<0.001, main treatment effects DD p<0.01.
(TIF)

Table S1 Gene specific primer sequences.
(DOC)
