# Supplementary figures and images for: 3T3-L1 Preadipocytes Exhibit Heightened Monocyte-Chemoattractant Protein-1 Response to Acute Fatty Acid Exposure
Source: PLoS One. 2014 Jun 9;9(6):e99382. doi: 10.1371/journal.pone.0099382 (PMC4049800; doi:10.1371/journal.pone.0099382)

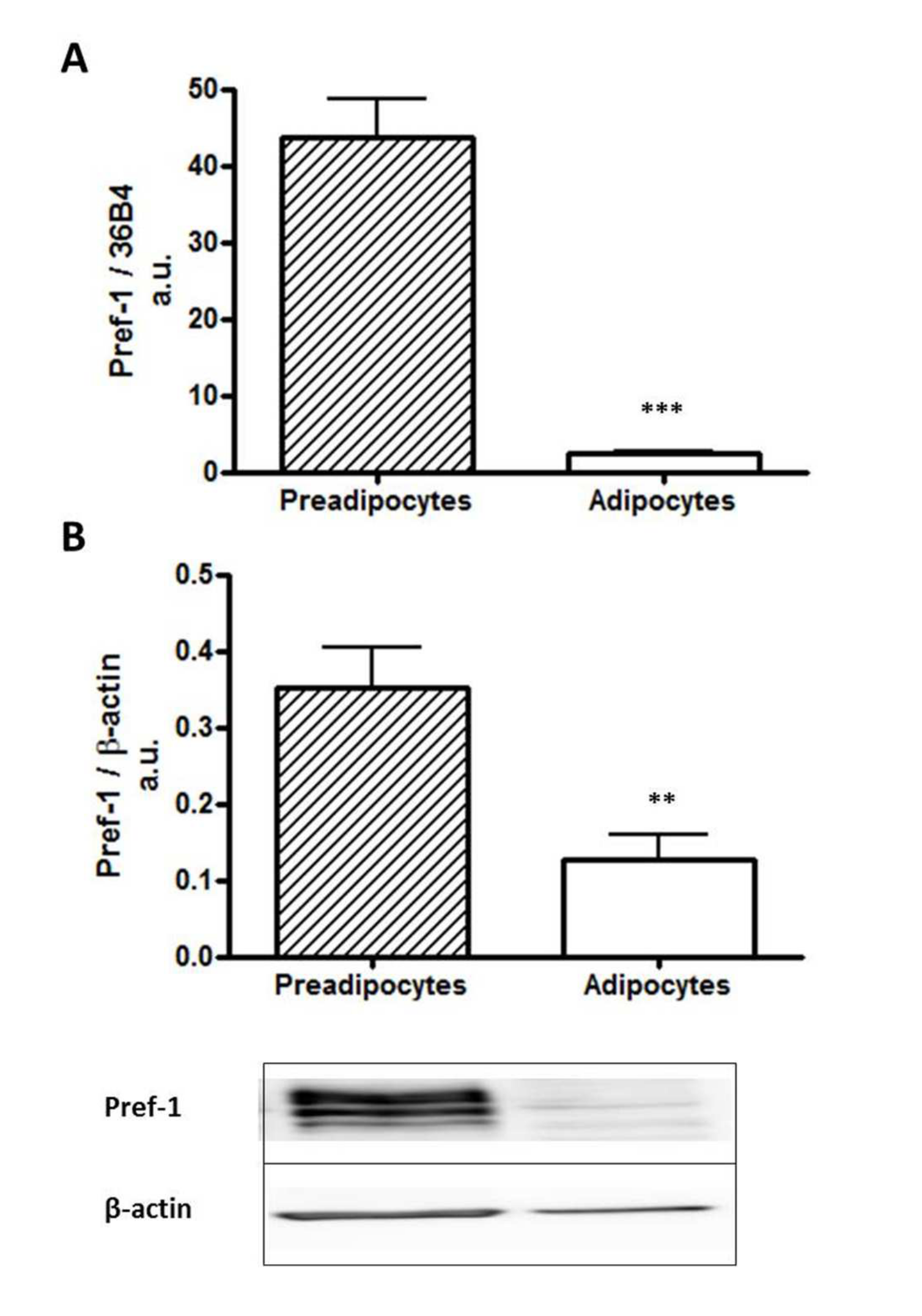

Supplement: Figure S1 — Preadipocyte factor-1 (pref-1) expression in 3T3-L1 preadipocytes and adipocytes. (A) mRNA and (B) protein expression of pref-1 in 3T3-L1 preadipocytes (2 days post-confluence) (hatched bars) and adipocytes (open bars) differentiated 6 days then maintained for a further 24 h (n = 5). ** p<0.01, *** p<0.001. (TIF) [file pone.0099382.s001.tif]

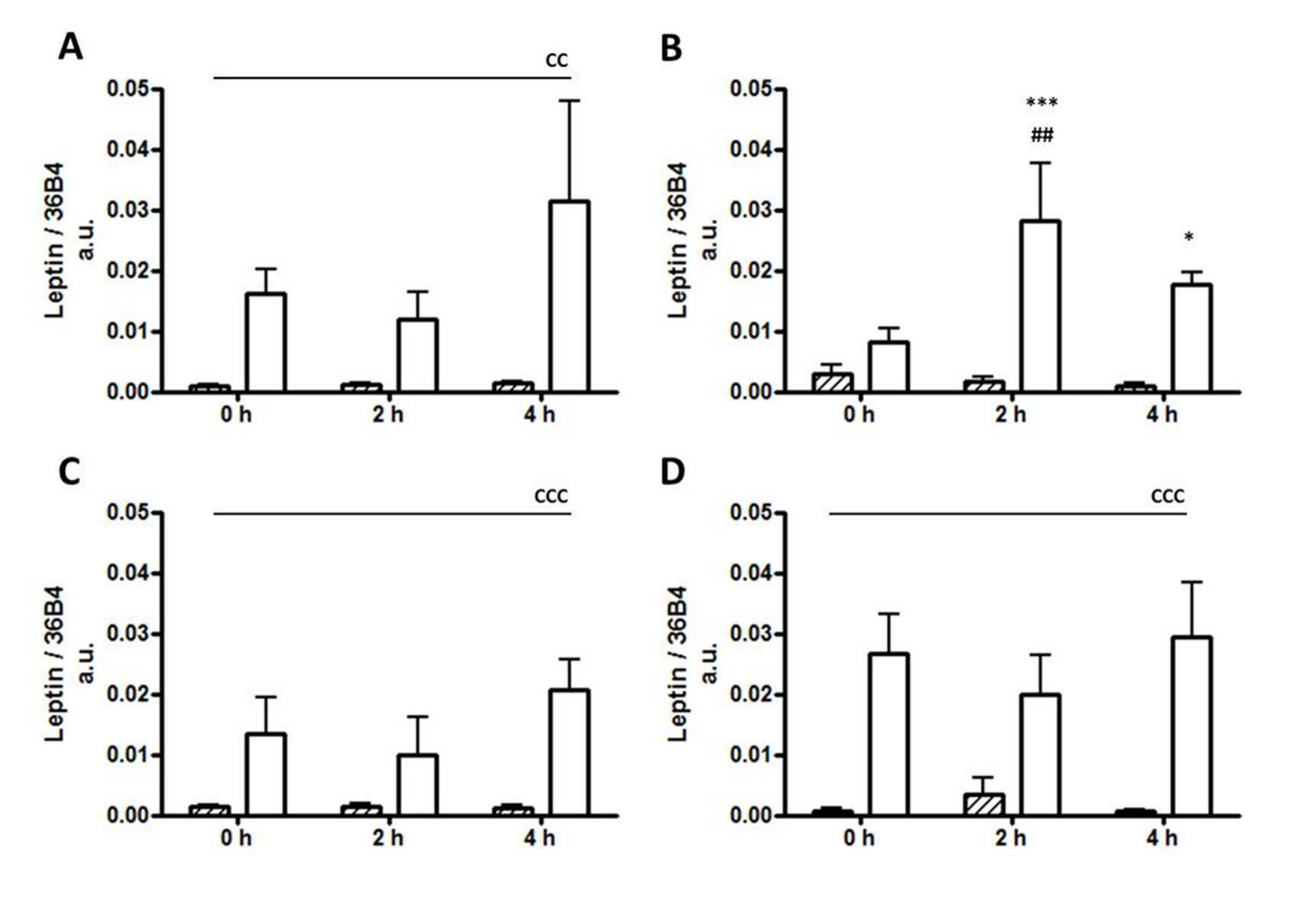

Supplement: Figure S2 — Leptin mRNA expression in 3T3-L1 preadipocytes and adipocytes. Leptin mRNA expression of 3T3-L1 preadipocytes (hatched bars) and adipocytes (open bars) treated with (A) LPS (10 ng/ml); (B) Palmitic acid (0.5 mM); (C) Myristic acid (0.5 mM); and (D) Oleic acid (0.5 mM) at 0, 2 and 4 h. Data are presented as mean ±SEM (n = 5) normalised to 36B4. * p<0.05, *** p<0.001 versus preadipocytes, ## p<0.01 versus 0 h. Main effects, Main cell type effect CC p<0.01, CCC p<0.001. (TIF) [file pone.0099382.s002.tif]

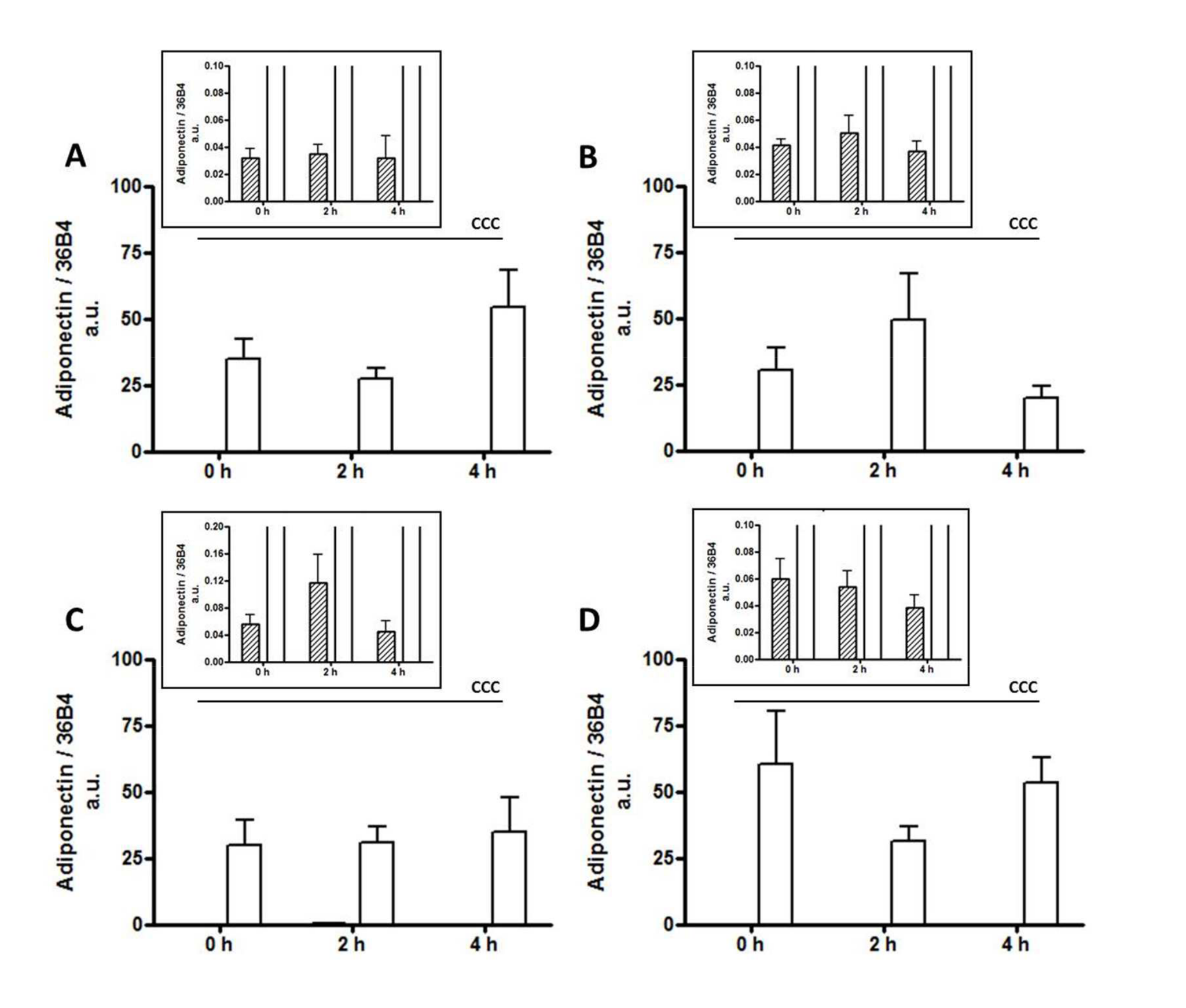

Supplement: Figure S3 — Adiponectin mRNA expression in 3T3-L1 preadipocytes and adipocytes. Adiponectin mRNA expression of 3T3-L1 preadipocytes (hatched bars) and adipocytes (open bars) treated with (A) LPS (10 ng/ml); (B) Palmitic acid (0.5 mM); (C) Myristic acid (0.5 mM); and (D) Oleic acid (0.5 mM) at 0, 2 and 4 h. Data are presented as mean ±SEM (n = 5) normalised to 36B4. Main cell type effect CCC p<0.001. (TIF) [file pone.0099382.s003.tif]

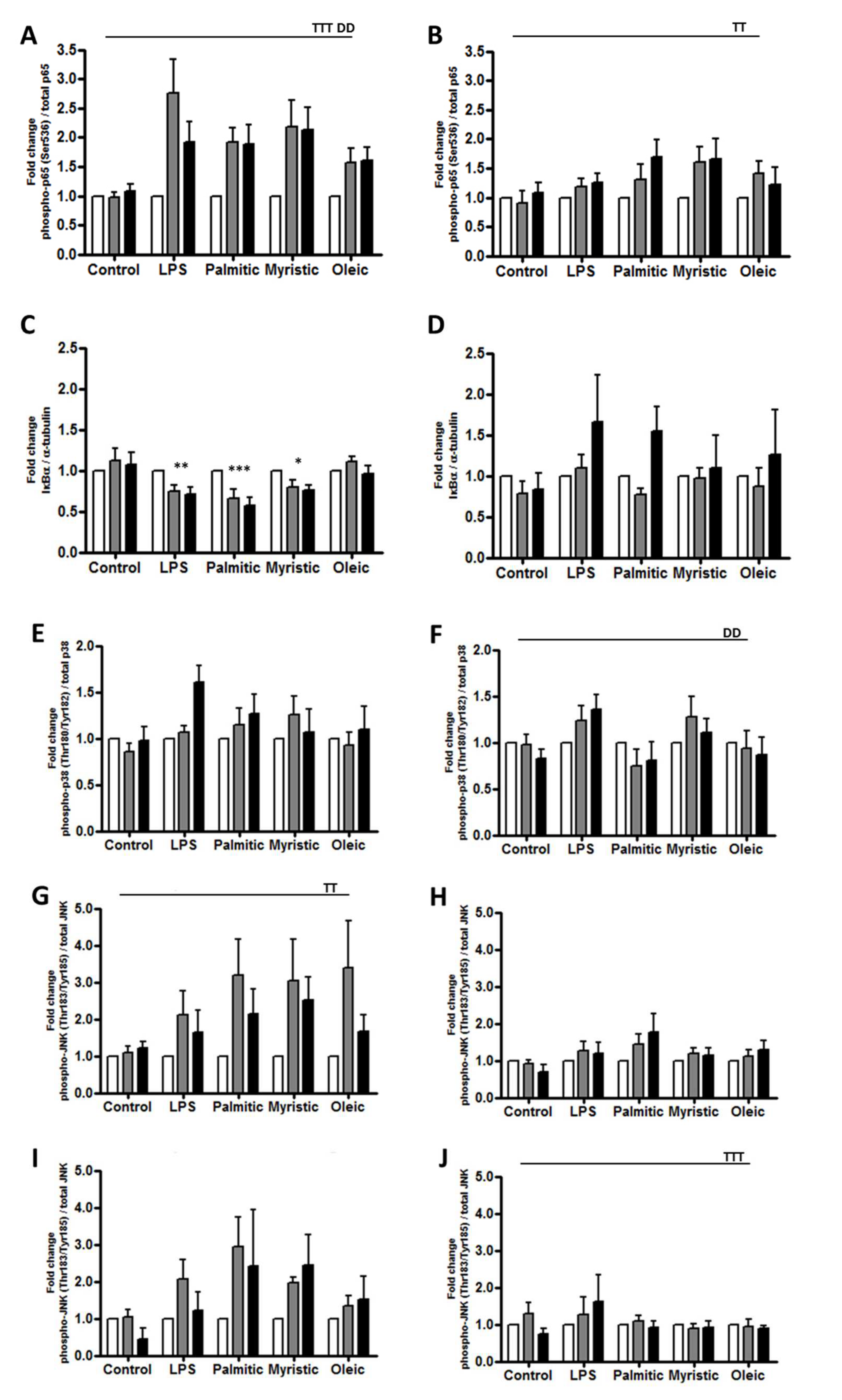

Supplement: Figure S4 — 3T3-L1 adipocytes were treated with LPS (10 ng/ml); Palmitic acid (0.5 mM); Myristic acid (0.5 mM); and Oleic acid (0.5 mM) for 0 (open bars), 1 (grey bars) and 2 h (black bars). Phosphorylation levels of p65 (Ser536) relative to total p65 and IκBα relative to α-tubulin in (A) and (C) preadipocytes and; (B) and (D) adipocytes, respectively. Phosphorylation levels of p38 MAPK (Thr180/Tyr182) relative to total p38 MAPK in (E) preadipocytes and (F) adipocytes. Phosphorylation levels of JNK (Thr183/Tyr185) relative to total JNK, p54 in (G) preadipocytes and (H) adipocytes and; p46 in (I) preadipocytes and (J) adipocytes, respectively as measured by Western blot analysis. Data are presented as fold change mean ±SEM (n = 5). Significant interactions, * p<0.05, ** p<0.01, *** p<0.001 versus control. Main time effects, TT p<0.01, TTT p<0.001, main treatment effects DD p<0.01. (TIF) [file pone.0099382.s004.tif]
