# Supplementary material for: 3T3-L1 Preadipocytes Exhibit Heightened Monocyte-Chemoattractant Protein-1 Response to Acute Fatty Acid Exposure
Source: PLoS One. 2014 Jun 9;9(6):e99382. doi: 10.1371/journal.pone.0099382 (PMC4049800; doi:10.1371/journal.pone.0099382)
Supplement: Table S1 — Gene specific primer sequences. (DOC) [file pone.0099382.s005.doc]

Supplementary data Table S1

| **Supplementary Table S1.** Primer sequences (mouse) | | | |
| --- | --- | --- | --- |
| **Gene** | **Accession No.** | **Forward** | **Reverse** |
| *36B4* | NM_007475.5 | GAGGAATCAGATGAGGATATGGGA | AAGCAGGCTGACTTGGTTGC |
| *Pref-1* | NM_010052.5 | GACCCACCCTGTGACCCC | CAGGCAGCTCGTGCACCCC |
| *MCP-1* | NM_011333.3 | CTTCCTCCACCACCATGCA | CCAGCCGGCAACTGTGA |
| *IL-6* | NM_031168.1 | ACAAGTCGGAGGCTTAATTACACAT | TTGCCATTGCACAACTCTTTTC |
| *TNF-α* | NM_013693.2 | atccgcgacctggaactg | ccgcctggagttctggaa |
| *Leptin* | NM_000230.2 | GTGTCGGTTCCTGTGGCTTT | TGGTCTTGATGAGGGTTTTGG |
| *Adiponectin* | NM_009605.4 | AAGGACAAGGCCGTTCTCT | TATGGGTAGTTGCAGTCAGTTGG |
